# Recent Advances in TiO_2_-Based Photocatalysis for the Treatment of Pesticide-Contaminated Wastewater: Mechanisms, Limitations, and Future Perspectives

**DOI:** 10.3390/ijms27125539

**Published:** 2026-06-18

**Authors:** Hieu Man Tran, Taeyoung Kim, Thi Huong Pham

**Affiliations:** Department of Materials Science and Engineering, Gachon University, 1342 Seongnam-daero, Sujeong-gu, Seongnam-si, Gyeonggi-do 13120, Republic of Korea

**Keywords:** pesticide residues, human health risk, environmental concerns, TiO_2_, photocatalysis

## Abstract

The discharge of pesticide residues (PRs) from agricultural activities into water bodies has raised concerns about their toxicity to humans and the ecosystem. Traditional methods such as adsorption, membrane filtration, biological treatment, and conventional filtration usually result in incomplete removal of PRs. Currently, removal of PRs using advanced oxidation processes, particularly metal oxide-based photocatalysts, is considered a promising way. This review provides a comprehensive overview of recent advances in the photocatalytic degradation of PRs using TiO_2_-based photocatalysts (T-BPs), the most widely investigated metal-oxide photocatalyst systems. First, we discuss the distribution, types, and negative impacts of major PRs on humans and the ecosystem. Next, we explore modification methods to enhance the properties of T-BPs, including light absorption behavior, charge separation rate, and photocatalytic degradation performance toward PRs. Afterward, this review carefully examines current challenges, such as complex water matrices, T-BP stability, energy supply for photocatalysis, and toxicity reduction. Finally, we highlight key future research directions, like the development of visible light-driven photocatalysts, enhanced mineralization efficiency, reduced secondary environmental risks, and the design of highly reliable catalyst and reactor systems for sustainable large-scale applications.

## 1. Introduction

Climate change and global warming have had negative impacts on all sectors, particularly agricultural production. The impact of heatwaves, droughts, and floods has significantly reduced agricultural output, raising concerns about global food security [1,2,3]. Besides, extreme weather has caused various diseases in crops and plants. Therefore, the use of pesticides and plant protection chemicals is increasing, leading to a large amount of these substances being released into the environment [2,4,5,6,7,8,9,10,11]. Table 1 lists the common pesticides typically detected in water. These compounds are classified into two main types, each with its specific application: herbicides and insecticides [2,3,6,7,11,12,13,14]. These compounds are often difficult to break down or decompose due to their chemical stability and can persist in the environment for extended periods [15,16]. This leads to their penetration into surface water, drinking water, and the human body through the food chain [16]. The discharge of pesticide pollutants can lead to water, soil, and air pollution, resulting in various problems for both humans and the environment. In particular, water pollution creates more serious impacts and poses risks to both ecosystems and public health [16]. Moreover, the loss of clean water and increasing water scarcity are becoming more serious each day, with over 2 billion people currently living in high-water-stress nations [17,18]. Hence, finding and developing technologies to reduce water pollution and reuse treated wastewater are urgent and necessary to protect water security and sustain our lives. Different methods have been developed to clean up water pollution, particularly for removing toxic organic contaminants such as PRs. For example, physical methods include adsorption (using activated carbon, biochar, zeolites) and membrane filtration (reverse osmosis, ultrafiltration, nanofiltration). On the other hand, chemical methods focus on advanced oxidation processes, which include ozonation, UV-H_2_O_2_, photo-Fenton, persulfate oxidation, and photocatalysis [19,20,21].

Advanced oxidation processes based on titanium dioxide are likely to be frequently used for removing PRs [5,22,23,24,25,26,27]. Previous studies have demonstrated that TiO_2_ offers multiple benefits, including high stability, non-toxicity, low cost, ease of scale-up, and high efficiency in UV light utilization. It can also be readily combined or modified with other compounds to create effective materials for the removal of organic pollutants, such as pesticide residues, under different environmental conditions [20,22,25,28,29]. Therefore, this study focuses on reviewing the removal of PRs using T-BPs. Firstly, this work examines the common pesticides used in agriculture and their effects on the environment and human health. Secondly, various methods for removing PRs from wastewater are identified. Then, we focus on the use of T-BPs to decompose the PRs from wastewater. The degradation performance and mechanisms of selected T-BPs for PR removal are discussed. Finally, this work investigates the benefits and limitations of using T-BPs for PRs removal and suggests some methods that could further improve the degradation performance of pesticide pollutants.

## 2. Environmental and Human Health Risks Associated with Pesticides

Common classes of pesticides and their main applications in agricultural activities are displayed in Table 2. As we know, pesticides are key in the agricultural sector because they can protect crops from various risks and help increase yields. Among the commonly used pesticides, five main types include herbicides, insecticides, nematicides, rodenticides, and fungicides [5,14,21,26,30,31,32,33]. For example, Glyphosate (C_3_H_8_NO_5_P, M = 169.07 g mol^−1^), Atrazine (C_8_H_14_ClN_5_, M = 215.68 g mol^−1^), and Paraquat (C_12_H_14_N_2_, M = 186.26 g mol^−1^) are used for weed control. Meanwhile, substances such as Chlorpyrifos (C_9_H_11_Cl_3_NO_3_PS, M = 350.59 g mol^−1^), Imidacloprid (C_9_H_11_ClN_5_O_2_, M = 255.66 g mol^−1^), Thiamethoxam (C_8_H_10_ClN_5_O_3_S, M = 291.71 g mol^−1^), Permethrin (C_21_H_20_Cl_2_O_3_, M = 391.29 g mol^−1^), and Cypermethrin (C_22_H_19_Cl_2_NO_3_, M = 416.31 g mol^−1^) are utilized to combat harmful insects.

Based on previous reports, global agricultural use of pesticides increased from 3.69 million metric tons in 2022 to 4.3 million metric tons in 2023 and may reach around 4.38 million metric tons by 2026. The use of large amounts of pesticides has led to the release of significant amounts of these substances into soil, water, and air, raising concerns about environmental pollution [4,6,7,8,9]. Moreover, due to their chemical stability, these compounds can persist for a long time in the environment and easily enter the human body through the food chain. In recent years, pesticide and plant protection drug residues exceeding the permissible threshold have been detected in some vegetables and fruits. In particular, this problem occurs more frequently and has become more serious in developing countries, where clean food remains a growing concern, and no reliable solution has yet been found. Therefore, the widespread presence of these substances in the environment poses significant risks to both ecosystems and human health [6,7,12].

**Figure 1:**
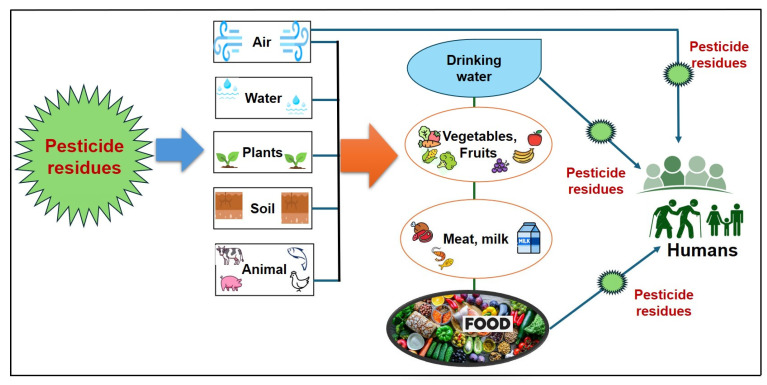
Major exposure pathways of PRs to human body.

Figure 1 shows the potential public health risks associated with human exposure to pesticide residues. As a result, PRs can enter the human body through various pathways, including food intake, drinking water, and inhalation of air pollution containing contaminants. For example, PRs on the surfaces of fruits, vegetables, and crops, as well as those accumulated in livestock and aquatic organisms (meat and fish), can be absorbed into the human body through the food chain. On the other hand, PRs can enter the ecosystem through agricultural runoff, in which pesticide residues pass through surface water and groundwater systems.

Due to their persistence, PRs may remain stable in water bodies for a while before entering plants and aquatic organisms, with a high likelihood of entering the human body through food. In addition, during pesticide spraying, some pesticides can evaporate, attach to dust particles, and spread into the air, where they may be inhaled by humans and cause harmful effects. Based on the findings, PRs can enter the environment and the human body through different pathways, with main concerns related to drinking water, food, and air. Moreover, due to their high toxicity and persistence, PRs can pose significant risks to ecosystems and human health. Therefore, we should develop suitable technologies to completely remove PRs, clean up the environment, and protect human health.

Table 3 summarizes the main impacts of different types of PRs on ecosystems and human health. As reported in previous studies, even at low concentrations, PRs have significant effects on developmental and metabolic processes. Also, some PRs are considered endocrine-disrupting chemicals and neurotoxic compounds [5,34,35,36]. For example, insecticides are commonly used to control insect populations, but organophosphates and neonicotinoids are highly toxic to both pests and non-target organisms (bees, butterflies, pollinators) [37,38]. Therefore, the decrease in these non-target organisms during the use of insecticides in agricultural activities may disrupt ecosystem balance.

In addition, long-term exposure to insecticides can negatively affect soil biota, including earthworms and beneficial microorganisms, thereby reducing soil health and disrupting nutrient cycling.

Herbicides are widely applied to control unwanted vegetation. However, certain herbicides, particularly glyphosate, may adversely affect non-target plant species. The increase in herbicide-resistant weeds has further worsened this issue, as they require larger amounts or stronger herbicides [35]. Moreover, the discharge of herbicides into aquatic environments can negatively affect aquatic organisms and animals. Over time, continuous herbicide application may reduce plant biodiversity, thereby disrupting food sources and habitats for animal species that depend on these plants.

Fungicides are widely used to control crop diseases, but they can negatively impact non-target organisms, particularly aquatic species. Many fungicides are highly toxic to fish and amphibians, leading to population decline and disruption of aquatic food webs. Using larger amounts of fungicide can also lead to the development of resistant fungal strains, which may require even stronger, more dangerous chemicals to control.

Rodenticides are widely used to control rodent pests, but they also pose risks to wildlife, particularly birds of prey such as owls and hawks. Therefore, PRs that remain in rodent pests can enter the food chain, poison wildlife, and cause significant negative effects. Moreover, the use of large amounts of rodenticides can disrupt and reduce the stability of the ecosystem.

Further, nematicides are used to control plant-parasitic nematodes, which also have various impacts on environmental and human health. For example, the release of nematicide residues into soil and aquatic environments can harm non-target soil organisms and aquatic species, leading to decreased soil quality and ecosystem damage. Therefore, all types of PRs, particularly nematicides, rodenticides, fungicides, herbicides, and insecticides, can pose significant risks to ecosystems and humans through both short-term and long-term toxic effects. For example, immediate toxicity may cause symptoms ranging from skin irritation and nausea to neurological disorders, seizures, and even death in agricultural workers or persons who have direct contact with these compounds. Moreover, long-term toxicity, even at low levels, can result in serious impacts such as neurodegenerative diseases, reproductive disorders, and even cancer. The remaining PRs on food become a major concern, especially for pregnant women and children. The exposure of pregnant women and children to some highly toxic PRs can lead to neurotoxic damage and other serious issues, such as birth defects and developmental delays.

**Figure 2:**
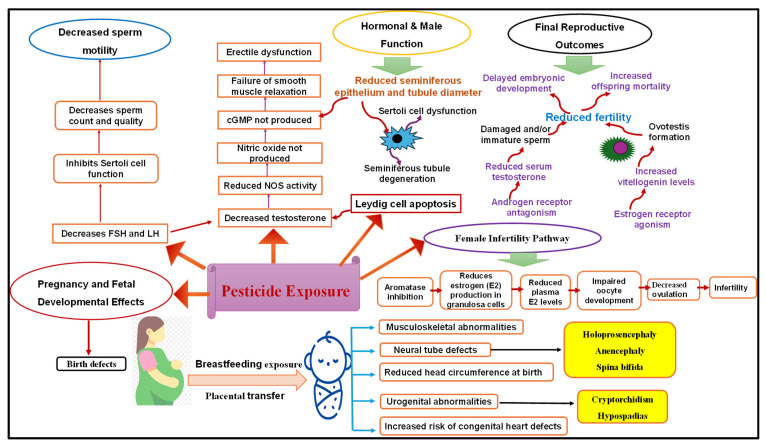
Mechanisms of pesticide toxicity in reproductive human health [39].

Figure 2 exhibits the effects of PRs on human health [39]. As observed, PRs may induce reproductive and developmental toxicity by disrupting key endocrine pathways. In addition, some PRs may affect embryonic and fetal development, leading to congenital abnormalities. The findings highlight that controlling and completely removing PRs are important for protecting ecosystems and human health.

## 3. Methods for Removal of PRs

Table 4 presents various methods for removing PRs from wastewater, including physical, biological, and chemical techniques. The results show that different treatment methods for removing PRs have distinct advantages and disadvantages. Besides, the evaluation of each method also depends on various factors such as PR removal efficiency, treatment cost, reusability, and so on [5,14,21,22,23,26,32,33].

For example, some methods that involve physical processes, such as adsorption, membrane filtration, and evaporation, do not require additional chemicals but can achieve good treatment efficiency. However, they have high operating costs and are applicable only to certain pesticides with high volatility and simpler molecular structures. In addition, the use of membranes to remove pesticide residues is associated with membrane fouling and disposal issues at the end of the treatment process. Other methods, such as solvent extraction, electrochemical treatment, and biosorption, also present some benefits, including high selectivity, effectiveness against persistent pesticides, and low environmental impact. However, several limitations have also been recognized, including the high cost of solvents, low performance, long processing times, and the need for an electrode. Moreover, researchers are more interested in using ozonation, photocatalysis, and the Photo-Fenton process for the treatment of PRs in wastewater. Additionally, the removal of PRs using photocatalysis displayed more benefits (low cost, high removal performance, easy scalability, and suitability for various types of pesticides) compared to ozonation and the Photo-Fenton process [5,14,40]. Therefore, we focus on evaluating the use of T-BPs for the degradation of PRs to improve understanding of the photocatalysis process and provide an effective method to mitigate the negative effects of hazardous pesticide pollution.

## 4. Removal of PRs in Wastewater Using TiO_2_ and T-BPs

### 4.1. Application of TiO_2_ and T-BPs

**Figure 3:**
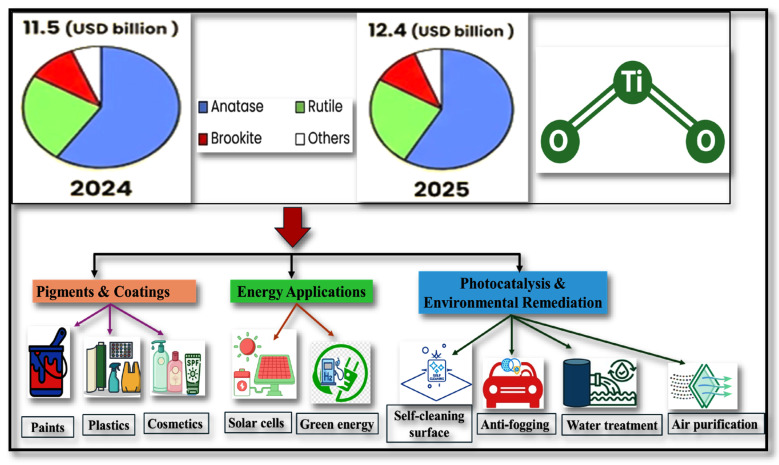
Main applications of TiO_2_.

Figure 3 shows that the global market for titanium dioxide continues to grow steadily (from about USD 11.5 billion in 2024 to USD 12.4 billion in 2025), confirming its role in various industries. For example, TiO_2_ has been widely utilized in the pigment, coating, energy, and environmental sectors, with pigment and coating applications accounting for the largest share of global usage [41]. TiO_2_ has high chemical stability, good refractive properties, strong light absorption, and non-toxicity, making it important for use in paints, plastics, paper, and cosmetics. Moreover, the use of TiO_2_ is increasing across the construction, automotive, and consumer products industries. On the other hand, TiO_2_ has become important in energy applications (solar cells, photocatalytic hydrogen production, and energy storage systems) [42] and environmental remediation (removal of organic pollutants, antibacterial applications, and self-cleaning) [22,43,44].

**Figure 4:**
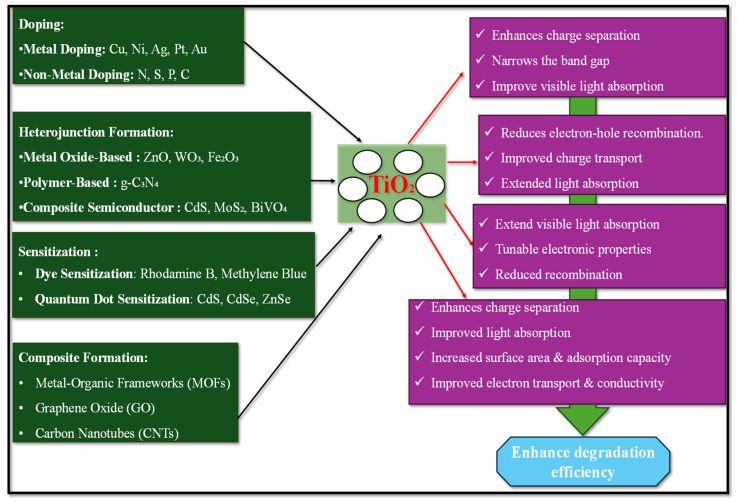
Benefits of different methods used to modify TiO_2_.

However, original or unmodified TiO_2_ still has some limitations, such as a wide band gap (3.2–3.3 eV), low activity under visible light, and a fast recombination rate of electron–hole pairs [19,20,22,25,28,29,45]. Therefore, various studies have focused on improving the photocatalytic activity of TiO_2_ through different methods, such as doping with metals (Cu, Ag, Pt, Au, Ni, Co) and non-metals (N, C, F, S, P), as well as combining with other catalysts/materials to form heterojunctions (g-C_3_N_4_, CQDs, GQDs, MXene, MOFs) [29,45,46]. Figure 4 summarizes different methods used to modify TiO_2_, focusing on three main objectives: (1) optimizing optical properties for improved solar light harvesting, (2) enhancing charge separation and transport, and (3) increasing the density of catalytically active sites. As a result, the modified TiO_2_-based catalysts can enhance light absorption, increase charge separation rates, and reduce the band gap energy, thereby improving photocatalytic efficiency. Thus, we can expand the application of T-BPs for sustainable energy production and environmental remediation with low cost and high efficiency.

### 4.2. Removal of PRs Using TiO_2_

As shown in Table 5, TiO_2_ photocatalyst effectively removes pesticide residues from aqueous solution under light-assisted conditions. For example, the removal efficiency of chlorpyrifos was 98% within 60 min, whereas diazinon was completely degraded within 30 min [47,48]. Endosulfan and imidacloprid also displayed high degradation efficiencies of 99% after 300 min and 90.2% after 180 min, respectively [49,50]. However, under visible light, the removal efficiency of chlorpyrifos (71–84%) was lower compared to UV irradiation [51,52]. Previous studies have demonstrated that TiO_2_ has a wide bandgap (3.2 eV) and is mainly activated by UV light rather than visible light. Moreover, the results in Table 5 indicate that high degradation efficiencies do not always lead to complete mineralization of PRs. For example, the degradation efficiency of diazinon was complete within 30 min, but TOC removal was only 42.2% at the same time [48]. Similarly, after 225 min of UV light exposure, the removal efficiency of cyproconazole was 85.8%, while TOC removal was limited to 38.5% [53]. The photodegradation of imidacloprid also shows the same trend, with only 68% TOC reduction after 180 min, while the removal efficiency reached 90.2% [50]. Under UVA irradiation, TiO_2_ can also effectively remove 98.8% of imidacloprid, 90.1% of thiamethoxam, and 92.0% of clothianidin. However, the TOC reductions were much lower, at 19.1%, 14.4%, and 14.1% for imidacloprid, thiamethoxam, and clothianidin, respectively [54]. Further, metribuzin required only 40 min for complete degradation, but it needed 300 min to achieve 80% TOC reduction [55]. These results indicate that photocatalytic processes typically proceed through various oxidation pathways, in which the original pollutants are rapidly transformed into intermediate products. However, the by-products require a long time to achieve complete mineralization, with a significantly slower rate. In some cases, mineralization may be incomplete, and the addition of treatment methods should be included.

From Table 5, it was observed that atrazine was completely degraded and almost fully mineralized (98.5% reduction in TOC after 20 min) under microwave-powered discharge lamps [56]. It highlights that, under strong oxidative conditions or high energy supply, persistent pesticides can be effectively broken down and transformed into inorganic substances. These results show that the degradation of PRs by TiO_2_ photocatalysis depends strongly on operating conditions, including the light source, light intensity, pollutant concentration, and the structure of the pesticides. It also displays that the degradation of PRs using TiO_2_ is always faster than the mineralization process. Therefore, to improve the conversion of PRs and their by-products into non-toxic inorganic substances, longer reaction times and additional treatment methods are needed.

**Table 5:** Photodegradation efficiency of PRs using TiO_2_.

Pesticide	Conditions	Degradation (%)	TOC Removal (%)	Refs.
Chlorpyrifos	UV light lamp (15 W UV lamp, 365 nm); Time: 1440 min; C_0_: 20 mg L^−1^.	81	NA	[57]
Chlorpyrifos	UV light (125 W mercury lamp, 80 W/m^2^); Time: 25 min; C_0_ = N/A.	90	NA	[58]
Chlorpyrifos	Visible light (300 W halogen lamp, 697 ± 5.33 lux, 7.5 × 10^−3^ W/m^2^); Time: 62.5 min; C_0_ = 2.74 mg L^−1^.	84	NA	[52]
Chlorpyrifos	Visible light (300 W halogen lamp, 697 ± 5.33 lux, 7.5 × 10^−3^ W/m^2^); Time: 55.15 min; C_0_ = 2.48 mg L^−1^.	71	NA	[51]
Chlorpyrifos	UV light (16 W mercury lamp, 251 nm); Time: 60 min; C_0_ = 10 mg L^−1^.	98	NA	[47]
Chlorpyrifos	UV light (9 W UV lamp, 254 nm); Time: 300 min; C_0_ = 5 mg L^−1^.	94	NA	[49]
Endosulphan	UV light (9 W UV lamp, 254 nm); Time: 300 min; C_0_ = 5 mg L^−1^.	99	NA	[49]
Diazinon	UV light (9 W mercury lamp, λ_max_ = 254 nm, 2.2 mW/cm^2^); Time: 30 min; C_0_ = 20 mg L^−1^.	100	42.2 (30 min)	[48]
Diazinon	UV light (125 W UV lamp, λ = 310–450 nm, 35–50 W/m^2^); C_0_ = 40 mg L^−1^.	99.6	NA	[59]
Quinalphos	UV light; Time: 240 min; C_0_ = 10 mg L^−1^.	89	100 (1440 min)	[60]
Imidacloprid	UV light (λ_max_ = 365 nm); Time: 100 min; C_0_ = 100 mg L^−1^.	57	NA	[61]
Imidacloprid	UVA (20 W mercury lamp, 315–400 nm, λ_max_ = 355 nm); Time = 120 min; C_0_ = 100 mg L^−1^.	98.8	19.1 (120 min)	[54]
Thiamethoxam	UVA (20 W mercury lamp, 315–400 nm, λ_max_ = 355 nm); Time = 120 min; C_0_ = 100 mg L^−1^.	90.1	14.4 (120 min)	[54]
Clothianidin	UVA (20 W mercury lamp, 315–400 nm, λ_max_ = 355 nm); Time = 120 min; C_0_ = 100 mg L^−1^.	92	14.1 (120 min)	[54]
Imidacloprid	UVC light (15 W UV lamp, λ_max_ = 254 nm, 17 W/m^2^); Time = 180 min; C_0_ = 20 mg L^−1^.	90.2	68 (180 min)	[50]
Cyproconazole	UV light (24 W UV lamp, λ_max_ = 365 nm photon flux = 38 ± 0.2 W/m^2^); Time = 255 min; C_0_ = 85 mg L^−1^.	85.8	38.5 (255 min)	[53]
Metobromuron	Sunlight (300 W Xe lamp, λ = 350–780 nm, 1.28 mW/cm^2^); Time: 40 min; C_0_ = 60.23 µM	96.2	NA	[62]
Metribuzin	Solar light (2200 W xenon arc lamp, >290 nm, 750 W/m^2^); Time = 40 min; C_0_ = 10 mg L^−1^.	100	80 (300 min)	[55]
Quinclorac	Solar light (1100 W xenon arc lamp, λ > 290 nm, 250 W/m^2^); Time: 40 min; C_0_ = 5 mg L^−1^/ultrapure water.	100	NA	[63]
Quinclorac	Solar light (1100 W xenon arc lamp, λ > 290 nm, 250 W/m^2^); Time: 130 min; C_0_ = 5 mg L^−1^/paddy field water.	98	NA	[63]
Atrazine	Light: NA; Time = 60 min; C_0_ = 200 mg L^−1^.	76.1	NA	[64]
Atrazine	UV light (microwave-powered electrodeless discharge lamps, EDLs, Hg vapor, λ_max_ = 254 nm); Time = 4 min; C_o_ = 20 mg L^−1^.	100	98.5 (20 min)	[56]
Quinalphos	UV light (UV lamp; 8 blue–black UV tubes, λ_max_ = 365 nm); Time: 180 min C_0_ = 20 mg L^−1^.	89.6	NA	[65]
Monocrotophos	UV light (UV lamp; 8 blue–black UV tubes, λ_max_ = 365 nm); Time: 180 min C_0_ = 25 mg L^−1^.	86.7	NA	[65]
Carbendazim	UV light (8 × 20 W black fluorescent lamps, light intensity 30 W m^−2^); Time: 60 min; C_0_ = 10 mg L^−1^.	96	85 (60 min)	[66]

NA: not available; TOC: Total Organic Carbon.

### 4.3. Degradation Mechanism of PRs by Using TiO_2_

**Figure 5:**
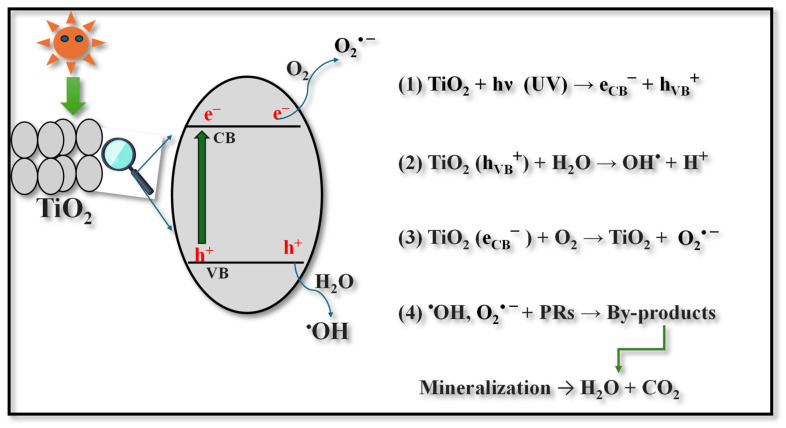
Degradation mechanism of PRs using TiO_2_.

Figure 5 shows the removal mechanism of pesticide residues via TiO_2_ photocatalysis. The process involves several steps: UV irradiation interacts with the TiO_2_ catalyst to generate electron–hole pairs, which then form reactive radicals that subsequently oxidize pesticide residues.

In detail, when TiO_2_ absorbs photons with energy equal to or greater than its bandgap, it becomes photoexcited, promoting electrons from the valence band to the conduction band and generating electron–hole pairs. Afterward, the electron-hole pairs move to the catalyst surface. There, they react with water and dissolved oxygen to form reactive oxygen species, mainly hydroxyl radicals and superoxide anions. Due to their high oxidation potential, these free radicals break complex organic bonds in PRs. This ongoing oxidative process leads to complete mineralization, converting intermediate by-products into harmless inorganic compounds such as carbon dioxide and water [56,57,59,62,64,67,68]. Therefore, the UV-driven TiO_2_ photocatalytic process can mineralize PRs into non-toxic compounds, hence markedly reducing related human health and environmental risks.

### 4.4. Removal of PRs Using T-BPs

To enhance the removal efficiency of PRs in the visible-light region, various methods have been used to modify TiO_2_ to form T-BPs. Based on the results shown in Table 6, it can be seen that the use of T-BPs has significantly increased the PRs degradation efficiency under solar, visible, and natural sunlight.

For example, the removal efficiencies of chlorpyrifos using CuO_2_/TiO_2_, TiO_2_/PANI, and CuO/TiO_2_/PANI were 60%, 82%, and 95%, respectively [69]. Moreover, the high degradation efficiencies of PRs using other composites, such as Zr^4+^-doped TiO_2_/CNTs (100% in 90 min under UV/visible light), GO–Fe_3_O_4_/TiO_2_ (97% in 60 min under visible light), and C,N-TiO_2_ (90% in 60 min under solar light), further confirm that the modification processes significantly improve the degradation performance of PRs by T-BPs [70,71]. On the other hand, In,S–TiO_2_@rGO photocatalyst shows complete degradation of atrazine and almost full mineralization (95.5% TOC removal) within 20 min under visible light [72]. The H_3_PW_12_O_40_/Ag–TiO_2_ can remove 98.6% atrazine within 60 min, but it takes 720 min to reach 89% TOC removal [73]. The presence of PMS or H_2_O_2_ in the photocatalytic process can rapidly increase the degradation performance of PRs due to the generation of additional reactive radicals from PMS and H_2_O_2_. For example, the TiO_2_@LaFeO_3_/PMS system completely removed atrazine in 120 min [74]. Similarly, the Fe/TiO_2_/H_2_O_2_ system also achieved 95% atrazine removal within 30 min under visible light [75]. Further, ZnO–TiO_2_/H_2_O_2_ completely removed diazinon within 120 min, confirming the effectiveness of combining photocatalysis with other treatment methods [76]. Hassan and co-workers developed Mo–TiO_2_/GO/MS composite as an effective material for the degradation of various pesticides, including endrin (94%), endosulfan (93.5%), heptachlor (90.7%), DDT (89%), endosulfan sulfate (88%), and aldrin (81.4%), within 20 min under UV light [77]. The results in Table 6 highlight the relationship between TiO_2_-based photocatalysts and PRs. The degradation performance of PRs improved with enhanced light absorption properties of photocatalysts, improved charge separation, and increased pollutant adsorption capacity. In addition, the results show a gap between PR degradation and TOC removal, similar to that observed with TiO_2_. T-BPs can quickly break down PRs into smaller by-products, while full mineralization takes much longer to break down PRs into CO_2_, H_2_O, and other inorganic compounds.

**Table 6:** Photocatalytic degradation performance of PRs using T-BPs.

Photocatalyst	Pesticide	Conditions	Degradation (%)	TOC Removal (%)	Refs.
CuO_2_/TiO_2_	Chlorpyrifos	Visible light (300 W tungsten lamp, λ > 420 nm); Time: 90 min C_0_ = 5 mg L^−1^.	60	NA	[69]
TiO_2_/PANI	Chlorpyrifos	Visible light (300 W tungsten lamp, λ > 420 nm); Time: 90 min C_0_ = 5 mg L^−1^.	82	NA	[69]
CuO/TiO_2_ /PANI	Chlorpyrifos	Visible light (300 W tungsten lamp, λ > 420 nm); Time: 90 min C_0_ = 5 mg L^−1^.	95	NA	[69]
Zr^4+^ doped TiO_2_/CNTs	Chlorpyrifos	Ultrasonic + UV/visible (400W Hg lamp); Time: 90min C_0_ = 30 mg L^−1^.	100	NA	[70]
GO-Fe_3_O_4_/TiO_2_	Chlorpyrifos	Visible light (300 W tungsten lamp); Time: 60 min; C_0_ = 10 mg L^−1^.	97	NA	[71]
GP-TiO_2_ -MnFe_2_O_4_	Atrazine	Pulsed discharge plasma (7 W); Time: 90 min; C_0_ = 10 mg L^−1^.	96.2	NA	[78]
N-TiO_2_	Atrazine	UVA light (2 × 15 W lamps, 2.47 ± 0.16 mW/cm^2^); Time: 90 min; C_0_ = 2.5 mg L^−1^.	45	NA	[79]
N-TiO_2_/ZSP	Atrazine	UV light (30 W UVA lamp, 2.47 ± 0.16 mW/cm^2^); Time: 90 min; C_0_ = 2.5 mg L^−1^.	94	NA	[79]
TiO_2_@LaFeO_3_/PMS	Atrazine	Visible light (200 W LED lamp, λ: 410–760 nm); Time: 120 min C_0_ = 2.5 mg L^−1^.	100	NA	[74]
Fe/TiO_2_/H_2_O_2_	Atrazine	Visible light (290 W, 400–600 nm, 30 W/m^2^); Time: 30 min; C_0_ = 10 mg L^−1^	95	NA	[75]
TiO_2_/Ozonation	Atrazine	[Ozone dose] = 10 mg min^−1^; Time: 30 min; C_0_ = 10 μM	93	56 (30 min)	[80]
In,S-TiO_2_@rGO	Atrazine	Visible light (300 W tungsten lamp); Time: 20 min; C_0_ = 20 mg L^−1^	100	95.5 (20 min)	[72]
H_3_PW_12_O_40_/Ag–TiO_2_	Atrazine	Solar light (300 W Xe lamp, 200 mW/cm^2^); Time: 60 min; C_0_ = 5 mg L^−1^.	98.6	89 (720 min)	[73]
TiO_2−x_/g-C_3_N_4_/CNFe	Atrazine	Visible light (300 W xenon lamp, λ > 420 nm, 200 mW/cm^2^); Time: 30 min C_0_ = 5 mg L^−1^.	85.9	33 (30 min)	[81]
TiO_2−x_/g-C_3_N_4_/CNFe /PMS	Atrazine	Visible light (300 W xenon lamp, λ > 420 nm, 200 mW/cm^2^); Time: 30 min C_0_ = 5 mg L^−1^.	95.4	47.8 (30 min)	[81]
TcPPCu/TiO_2_	Atrazine	Visible light (125 W xenon lamp, λ > 420 nm); Time: 60 min; C_0_ = 20 mg L^−1^.	82	NA	[68]
W-TiO_2_/clay	Atrazine	Solar light (300 W Xe lamp, 0.47 mW/cm^2^); Time: 240 min; C_0_ = 2.5 mg L^−1^.	90	NA	[82]
C, N-TiO_2_	Diazinon	Solar light (xenon lamp, 80–100 kLux); Time: 480 min C_0_ = 4 mg L^−1^.	88.5	60 (480 min)	[83]
C, N-TiO_2_	Chlorpyrifos	Solar light (90–110 kLux); Time: 60 min C_0_ = 8 mg L^−1^.	90	62.1 (60 min)	[45]
Cu-doped TiO_2_/GO	Chlorpyrifos	UV light, Time: 80 min C_0_ = 100 mg L^−1^.	91.4	NA	[29]
Cu-doped TiO_2_/GO	Chlorpyrifos	Visible light, Time: 80 min C_0_ = 100 mg L^−1^.	78.2	NA	[29]
Mo –TiO_2_/ GO/MS	Endrin	UV light (150 W xenon arc lamp, 365 nm); Time: 20 min C_0_ = 4.5 mg L^−1^.	94	NA	[77]
Mo –TiO_2_/ GO/MS	Aldrin	UV light (150 W xenon arc lamp, 365 nm); Time: 20 min C_0_ = 24.4 mg L^−1^.	81.4	NA	[77]
Mo –TiO_2_/ GO/MS	Heptachlor	UV light (150 W xenon arc lamp, 365 nm); Time: 20 min C_0_ = 12.2 mg L^−1^.	90.7	NA	[77]
Mo –TiO_2_/ GO/MS	Endosulfan sulfate	UV light (150 W xenon arc lamp, 365 nm); Time: 20 min C_0_ = 1.3 mg L^−1^.	88	NA	[77]
Mo –TiO_2_/ GO/MS	DDT	UV light (150 W xenon arc lamp, 365 nm); Time: 20 min C_0_ = 47.4 mg L^−1^.	89	NA	[77]
Mo –TiO_2_/ GO/MS	Endosulfan	UV light (150 W xenon arc lamp, 365 nm); Time: 20 min C_0_ = 13.8 mg L^−1^.	93.5	NA	[77]
TiO_2_@g-C_3_N_4_	Malathion	UV light (8 W UVB lamp, 306 nm); Time: 150 C_0_ = 20 mg L^−1^.	74	NA	[84]
Fe_3_O_4_@SiO_2_@mTiO_2_	Acephate	UV light (100 W mercury lamp, λmax = 365 nm); Time: 60 min C_0_ = 100 mg L^−1^.	100	78	[85]
Fe_3_O_4_@SiO_2_@mTiO_2_	Omethoate	UV light (100 W mercury lamp, λ_max_ = 365 nm); Time: 45 min C_0_ = 100 mg L^−1^.	100	75	[85]
Fe_3_O_4_@SiO_2_@mTiO_2_	Methyl parathion	UV light (100 W mercury lamp, λmax = 365 nm); Time: 60 min C_0_ = 100 mg L^−1^.	100	85	[85]
TiO_2_/Fe_2_O_3_	Diazinon	Visible light (60 W visible lamp, light intensity = 14 W cm^−2^); Time: 45 min C_0_ = 10 mg L^−1^.	95	NA	[86]
Pt-TiO_2_	Diazinon	UV light (990 W Xe lamp, light intensity = 1.5 mW cm^−2^); Time: 30 min C_0_ = 30 mg L^−1^.	100	88 (1800 min)	[67]
ZnO-TiO_2_/H_2_O_2_	Diazinon	UV light, (125 W UV lamp, Time: 120 min) C_0_ = 20 mg L^−1^.	100	NA	[76]
2% WO_3_/TiO_2_	Malathion	Natural sunlight (solar intensity ~1030 W m^−2^, exposure time 11:00–16:00 h; UV dose = 180 kJ m^−2^); Time: 120 min; C_0_ = 12 mg L^−1^.	99	76 (300 min)	[87]
GO@TiO_2_	Imidacloprid	UV light (UV lamp, λ_max_ = 365 nm); Time: 30 min; C_0_ = 100 mg L^−1^.	93	NA	[61]

NA: not available; TOC: Total Organic Carbon.

### 4.5. Degradation Mechanisms of PRs Using T-BPs

**Figure 6:**
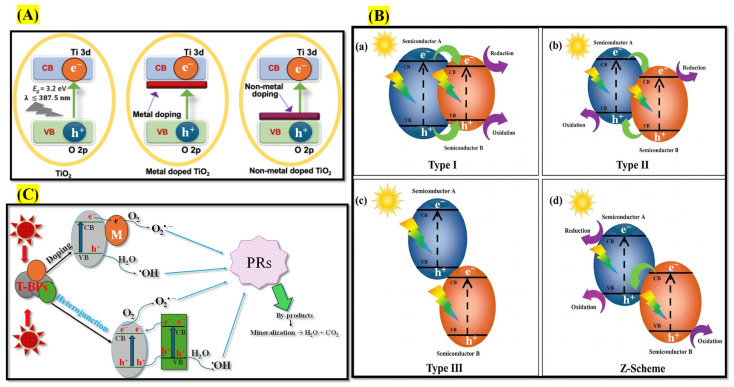
(**A**): Effects of metal and non-metal dopants on the properties and photocatalytic activity of TiO_2_-based photocatalysts. Reproduced from [89]; (**B**): Schematic diagram of electron–hole pair separation in (**a**) Type I, (**b**) Type II, (**c**) Type III, and (**d**) Z-scheme heterojunctions. Reproduced from [92]; (**C**): Degradation mechanisms of PRs using T-BPs.

Figure 6A shows the effect of metal and non-metal doping on the photocatalytic activity of TiO_2_. Different metal dopants can improve the photocatalytic performance of TiO_2_ in different ways. For example, Co, Ni, and Cu can form Ti^3+^ centers and oxygen vacancies, improving defect-mediated activity. Pt, Au, and Ag enhance electron transfer and suppress electron–hole recombination, while La and Ce act as electron traps and may help increase the catalyst surface area [88,89]. Moreover, non-metal doping (such as N, C, S, and P) is another effective method to improve the visible-light activity of TiO_2_ [89]. It introduces new energy states above the valence band and narrows the band gap, which enhances light absorption and photocatalytic performance. For example, Belver and co-workers used W-doped Titanium dioxide to remove Atrazine. They found that W-doped TiO_2_ showed a slight shift in light absorption from 390 to 405 nm and a reduced band gap from 3.2 to 2.96 eV [82]. In addition, W doping improved photocatalytic activity by creating new energy levels and charge-trapping sites, thereby reducing electron–hole recombination and enhancing charge separation, thereby improving PRs degradation [82]. On the other hand, Mohamad Idris and co-workers demonstrated that co-doping with C and N also reduces the band gap energy of Titanium dioxide from 3.2 to 2.95 eV [83]. This reduction in band gap can improve visible-light absorption and enhance the photocatalytic performance of C, N-TiO_2_ during PRs degradation under solar light irradiation [83].

Figure 6B displays different heterojunction structures used to improve the photocatalytic activities of TiO_2_. To improve the photocatalytic performance of TiO_2_, heterojunction structures have been widely developed to enhance charge separation and light absorption [89,90]. Among heterojunctions, Type II systems are widely studied because they can significantly reduce electron-hole recombination, thereby generating more reactive radicals and improving the degradation performance of PRs. They can also extend light absorption into the visible range, further reducing operating costs by using natural sunlight.

Further, Z-scheme heterojunctions have also received much attention recently. The formation of Z-scheme structures provides strong oxidation and reduction capabilities while also improving charge separation rates. In this process, electrons from one semiconductor recombine with holes from the other, leaving highly active electrons and holes on opposite sides. This allows the catalyst to maintain strong redox power and generate more reactive species. It can also extend visible-light absorption and improve the degradation efficiency of PRs [90].

For example, Amanah and co-workers prepared a TiO_2_/g-C_3_N_4_ Z-scheme heterostructure, which showed significant improvement in the photocatalytic degradation of Malathion [84]. The synthesized material not only enhanced visible-light absorption but also improved charge separation at the heterojunction. In this system, electrons in the conduction band (CB) of g-C_3_N_4_ were transferred to the CB of TiO_2_, reducing electron–hole recombination and improving photocatalytic efficiency. As a result, more reactive oxygen species (ROS), such as O_2_•^−^ and •OH, were generated. These reactive species played an important role in the degradation of malathion into intermediate products and eventually mineralized it into CO_2_ and H_2_O. Meanwhile, holes in the valence band (VB) of TiO_2_ could react with H_2_O or OH^−^ to produce •OH radicals, while the accumulated electrons in the CB participated in the formation of O_2_•^−^, further supporting the photocatalytic degradation process [84].

Figure 6C summarizes the mechanism for PRs removal using T-BPs, which is similar to the process of pesticide pollutant removal by TiO_2_ in both the initial stage (catalyst activation by light) and the final stage (pollutant decomposition by free radicals). However, the intermediate steps, including electron-hole separation and free radical formation, differ depending on the method used to modify TiO_2_. The modifications affect the photocatalytic process by altering charge separation, reactive oxygen species generation, and the overall catalytic activity of TiO_2_. It shows how doping and combining TiO_2_ with other catalysts can enhance the photo-catalytic degradation of PRs under solar, visible, and sunlight conditions [29,65,70,71,74,75,77,78,79,82,84,85,87,91]. As a result, doping can create an electron acceptor, while combining it with other catalysts can provide a new charge transport pathway, thereby increasing the efficiency of electron-hole separation. Enhancing electron–hole separation efficiency increases free radical generation, thereby improving PR degradation efficiency.

## 5. Benefits and Challenges of Using T-BPs for PRs Removal

### 5.1. Benefits of Using T-BPs

The use of T-BPs is highly effective in removing pesticide residues and offers a promising technology to address environmental and human health risks caused by PRs [5,7,93]. Even under natural sunlight, some T-BPs showed high removal performance of PRs, nearly 100%, confirming the potential for utilizing these materials in environmental remediation. This highlights the ability of T-BPs to use renewable energy with no initial cost for the treatment of PRs in wastewater [69,71,94]. Moreover, the photodegradation of PRs using T-BPs involves mineralization, in which the pollutants and their intermediates are broken down into non-toxic inorganic compounds, such as carbon dioxide and water. This is the most important benefit of the photocatalysis process, as it can transform the original pollutants into non-toxic compounds with the support of light. In addition, this process does not produce secondary waste like other methods (adsorption, biological), making T-BPs an effective solution for treating PRs in water and soil.

### 5.2. Challenges of Using T-BPs

Despite the benefits of using T-BPs to remove PRs, challenges still remain that need further study and development. For example, altering the optical properties of TiO_2_ via methods such as doping or combining with other catalysts can increase material costs, thereby raising the cost of wastewater treatment. Furthermore, sunlight is not always available due to weather and seasonal changes, which may affect the continuous operation of wastewater treatment systems. In addition, T-BPs primarily exist in powder form, which can bring significant challenges to the efficient recovery and reuse of photocatalysts. In general, the powder is difficult to separate from the treated water, leading to the loss of T-BPs during the recycling process and decreasing the removal efficiency of PRs [21,26,46].

Table 7 summarizes the main challenges that remain during the treatment of pesticides in wastewater using T-BPs.

To limit challenges in pesticide removal, further development of T-BPs should be pursued. The development needs to focus on enhancing efficiency, stability, and cost-effectiveness to be applicable in a wide range of environmental conditions and practical situations. In general, the real wastewater is often significantly diminished due to the complex composition of actual effluents, which include competing ions, natural organic matter, suspended solids, and other contaminants. These components reduce light penetration, block active sites, and consume reactive species. As a result, scaling up from laboratory to full-scale treatment systems remains challenging. Therefore, we should develop a suitable reactor that fully contacts the light source and increases interaction between the wastewater and T-BPs. Also, enhancing the reusability of T-BPs is important to reduce operational costs and support scale-up applications.

## 6. Conclusions and Future Perspectives

This review highlights effective methods for removing pesticide residues from wastewater using advanced oxidation processes based on TiO_2_ photocatalysts. Removal of PRs using T-BPs offers various benefits for environmental cleanup and helps mitigate risks to human health. This method can decompose pesticides into intermediate products, which are then mineralized into non-harmful substances, reducing their negative effects and long-term exposure to humans. The treatment of PRs using T-BPs also offers additional advantages over other methods, such as adsorption, biological processes, membrane filtration, ozonation, extraction, and evaporation. According to the literature review, T-BPs can remove various pesticides with high degradation efficiency, up to 100%. Additionally, the light source is a key factor that directly affects the removal efficiency of PRs by T-BPs. TiO_2_ mostly works under UV light, while modifying it through doping or combining it with other materials can harness visible light and natural sunlight to activate the photocatalytic reaction. Additionally, the degradation mechanisms of PRs using TiO_2_ and T-BPs are discussed to highlight the main differences between the original and modified catalysts. In addition, the benefits and remaining issues are highlighted to further develop T-BPs and improve their use in the practical application of removing PRs.

Based on previous studies, the removal of PRs has been widely evaluated in aqueous solutions and with single pollutants. Therefore, future research should focus on the removal of PRs using T-BPs in real wastewater samples or the degradation of mixed pesticide pollutants. Moreover, the degradation of PRs may produce more toxic by-products if mineralization is not complete. Thus, future research to investigate by-products and their toxicity is necessary. On the other hand, to reduce the treatment cost of PRs, the utilization of natural sunlight should be considered. Also, future research should focus on kinetic studies to provide important information for reactor design, process optimization, and scale-up of TiO_2_-based photocatalytic systems for the removal of pesticide residues from wastewater in real applications. In addition, with the increasing use of AI, future research should also focus on integrating machine learning approaches to model pollutant degradation. By applying different AI tools, we may predict by-product formation and optimize other environmental factors to maximize the removal efficiency of PRs.

## Figures and Tables

**Table 1:** List of common pesticides frequently detected in water sources.

Pesticide	Chemical Formula	Structure	Group
Atrazine	C_8_H_14_ClN_5_	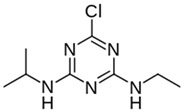	Herbicide
Glyphosate	C_3_H_8_NO_5_P	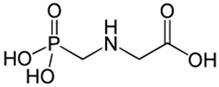	Herbicide
Chlorpyrifos	C_9_H_11_Cl_3_NO_3_PS	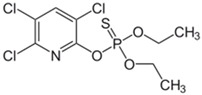	Insecticide
Malathion	C_10_H_19_O_6_PS_2_	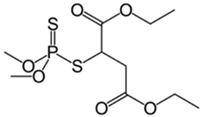	Insecticide
Diazinon	C_12_H_21_N_2_O_3_PS	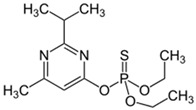	Insecticide
Simazine	C_7_H_12_ClN_5_	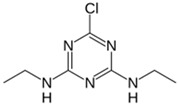	Herbicide
Imidacloprid	C_9_H_10_ClN_5_O_2_	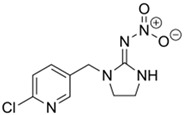	Insecticide
Carbaryl	C_12_H_11_NO_2_	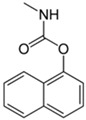	Insecticide
Pendimethalin	C_13_H_19_N_3_O_4_	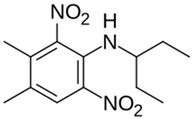	Herbicide
Endosulfan	C_9_H_6_Cl_6_O_3_S	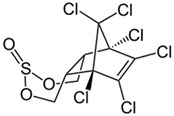	Insecticide
Trifluralin	C_13_H_16_F_3_N_3_O_4_	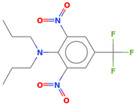	Herbicide
Bifenthrin	C_23_H_22_ClF_3_O_2_	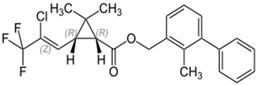	Insecticide
2,4-Dichlorophenoxyacetic acid	C_8_H_6_Cl_2_O_3_	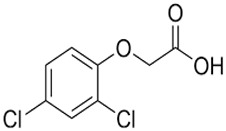	Herbicide

**Table 2:** List of common pesticides and their applications.

Pesticides	Application
Herbicides: Glyphosate (C_3_H_8_NO_5_P, M = 169.07 g mol^−1^), Atrazine (C_8_H_14_ClN_5_, M = 215.68 g mol^−1^), and Paraquat (C_12_H_14_N_2_, M = 186.26 g mol^−1^).	- Remove unwanted weeds
Insecticides: Chlorpyrifos (C_9_H_11_Cl_3_NO_3_PS, M = 350.59 g mol^−1^), Imidacloprid (C_9_H_11_ClN_5_O_2_, M = 255.66 g mol^−1^), Thiamethoxam (C_8_H_10_ClN_5_O_3_S, M = 291.71 g mol^−1^), Permethrin (C_21_H_20_Cl_2_O_3_, M = 391.29 g mol^−1^), Cypermethrin (C_22_H_19_Cl_2_NO_3_, M = 416.31 g mol^−1^)	- Control harmful insects
Nematicides: Fenamiphos (C_13_H_22_NO_3_PS, M = 303.36 g mol^−1^), 1,3-Dichloropropene (C_3_H_4_Cl_2_, M = 110.97 g mol^−1^), and Oxamyl (C_7_H_13_Cl_3_N_3_O_3_, M = 175.2 g mol^−1^)	- Control microscopic worms
Fungicides: Mancozeb (C_4_H_6_N_2_S_4_Mn·C_4_H_6_N_2_S_4_Zn, M = 541.2 g mol^−1^), Copper Sulfate (CuSO_4_, M = 159.61 g mol^−1^)	- Prevent or treat fungal infections in crops.
Rodenticides: Brodifacoum (C_31_H_23_BrO_3_, M = 523.32 g mol^−1^), Warfarin (C_19_H_16_O_4_, M = 308.33 g mol^−1^) and Zinc Phosphide (Zn_3_P_2_, M = 258.11 g mol^−1^)	- Control rats and mice

**Table 3:** Environmental and human health effects associated with PRs.

Classes of Pesticides	Environmental Impacts	Human Health Impacts
**Insecticides**	1. Soil contamination - Persist in soil, disrupting microbiomes - Reducing fertility - Harmful to beneficial organisms 2. Water pollution - Discharging and causing pollution to groundwater and surface water - Harmful to fish and aquatic organisms 3. Air pollution - Entering the air through the spraying process and reducing air quality 4. Biodiversity loss - Disrupts ecosystems	1. Acute Effects - Dizziness - Headaches - Skin irritation - Death 2. Chronic effects - Reduce memory - Cancer risk - Endocrine disruption - Impact on hormones - Birth defects
**Herbicides**	1. Soil contamination - Reduce the soil quality - Effect on microorganisms 2. Water pollution - Harmful to aquatic life - Impacts on non-target plants 3. Biodiversity loss - Disrupting ecosystems	1. Skin irritation 2. Chronic - Hormonal disruption - Reproductive problem - Potential risk of cancer 3. Neurological effects - Reducing brain function.
**Fungicides**	1. Soil impact - Decreasing soil quality - Harmful to microorganisms 2. Water contamination - Effect on quality of groundwater and surface water 3. Biodiversity loss - Imbalanced ecosystems	1. Acute - Skin irritation - Eye irritation - Respiratory problem 2. Chronic - Potential risks of cancer - Endocrine disruption issue 3. Neurological impact - Neurotoxicity
**Rodenticides**	1. Soil contamination - Reduce soil quality 2. Water pollution - Reduce water quality - Harmful to aquatic life 3. Non-target species - Negative impact on wildlife (birds, mammals) 4. Biodiversity impact - Destroy the ecosystem balance.	1. Acute - Dizziness - Headaches 2. Chronic - Liver damage - Kidney damage - Cancer risks 3. Poisoning - Death
**Nematicides**	1. Soil impact - Harm to earthworms - Reduce soil quality 2. Water contamination - Reduce water quality - Harm to aquatic life 3. Non-target species - Harm to wildlife 4. Biodiversity loss - Destroy ecological balance	1. Acute - Skin irritation, nausea - Dizziness 2. Chronic - Liver damage - Reproductive toxicity - Cancer risks. 3. Poisoning - Contribute to serious health issues or death

**Table 4:** Comparative evaluation of common technologies for PR removal.

Methods	Advantages	Disadvantages
Membrane	High removal efficiency, suitable for various contaminants	Membrane fouling, high operational cost
Adsorption	Cost-effective, widely available	Limited by adsorbent capacity, requires regeneration
Electrochemical	Effective for persistent pesticides, no additional chemicals	High energy consumption requires electrode
Ozonation	Strong oxidation power, effective against resistant compounds	Formation of toxic byproducts requires control.
Photocatalysis	Environmentally friendly Utilizes solar energy-reducing energy costs Effective degradation of pesticides. Suitable for both lab-scale and pilot-scale applications	Requires a catalyst Requires light source Catalyst deactivation over time
Photo-Fenton	Highly effective in degrading pesticides, no harmful residues	High operational costs, Difficult to implement on a large industrial scale

**Table 7:** Key challenges in T-BPs for real wastewater treatment.

Challenges	Main Effects	Results
Natural organic matter (Humic and fulvic acids)	- Reduce generation of reactive radicals. - Competitive and blocked active sites - Absorbs and reduces the intensity of incident light in water.	- Decrease the photodegradation efficiency of pesticides
Dissolved inorganic ions (Metal ions, Cl^−^ CO_3_^2−^, SO_4_^2−^)	- Reduce the amount of primary radicals - Blocked active sites - Transform into secondary radicals.	- Decrease the removal efficiency of pesticides - Possibly form toxic by-products
Suspended solids and turbidity	- Reduce light transmission - Decrease interaction between the catalyst and the light - Inhibit the production of radicals	- Decrease the removal efficiency of pesticides
Light sources	- UV, Solar, visible lights: High energy consumption - Natural sunlight: Difficult to control	- High operation cost - Inconsistent treatment performance
Co-existing pollutants/compounds	- Catalyst deactivation	- Reduce the removal efficiency of target pollutants
Solid waste after the treatment process	- Difficult to separate from treated wastewater - Leaching into the environment - Sludge and regeneration issues	- Increase operation costs - Can cause secondary pollution - Need additional methods to control solid waste/sludge

## Data Availability

The original contributions presented in this study are included in the article. Further inquiries can be directed to the corresponding author.
